# Assessing the Compressive and Impact Behavior of Plastic Safety Toe Caps through Computational Modelling

**DOI:** 10.3390/polym13244332

**Published:** 2021-12-10

**Authors:** Pedro Veiga Rodrigues, Bruno Ramoa, Ana Vera Machado, Philip Cardiff, João Miguel Nóbrega

**Affiliations:** 1 Department of Polymer Engineering, Institute for Polymers and Composites (IPC), Campus de Azurém, University of Minho, 4804-533 Guimarães, Portugal; bruno.ramoa@dep.uminho.pt (B.R.); avm@dep.uminho.pt (A.V.M.); mnobrega@dep.uminho.pt (J.M.N.); 2 School of Mechanical and Materials Engineering, University College Dublin, Belfield, D04 V1W8 Dublin, Ireland; philip.cardiff@ucd.ie

**Keywords:** toe cap, structural analysis, safety footwear, FVM, OpenFOAM

## Abstract

Toe caps are one of the most important components in safety footwear, but have a significant contribution to the weight of the shoe. Efforts have been made to replace steel toe caps by polymeric ones, since they are lighter, insulated and insensitive to magnetic fields. Nevertheless, polymeric solutions require larger volumes, which has a negative impact on the shoe’s aesthetics. Therefore, safety footwear manufacturers are pursuing the development of an easy, low-cost and reliable solution to optimize this component. In this work, a solid mechanics toolbox built in the open-source computational library, OpenFOAM^®^, was used to simulate two laboratory standard tests (15 kN compression and 200 J impact tests). To model the polymeric material behavior, a neo-Hookean hyper-elasto-plastic material law with J2 plastic criteria was employed. A commercially available plastic toe cap was characterized, and the collected data was used for assessment purposes. Close agreements, between experimental and simulated values, were achieved for both tests, with an approximate error of 5.4% and 6.8% for the displacement value in compression and impact test simulations, respectively. The results clearly demonstrate that the employed open-source finite volume computational models offer reliable results and can support the design of toe caps for the R&D footwear industry.

## 1. Introduction

### 1.1. Safety Footwear

Worker safety is of primary concern inside the workplace. According to Eurostat, since 2010, the total number of accidents in the workplace exceeds 3 million per year, in Europe [1]. From these, more than 28% were in the lower extremities of industrial workers, which are the most prone group to this kind of injury. Employers from industrialized countries are required to provide personal protective equipment (PPE), to help mitigating any work-related injuries. PPE for safety footwear is available in three different standardized categories: safety shoes, protective shoes and occupational shoes, Table 1 [2,3,4]. They mainly differ in the protection level provided to the wearer through the use of a toe cap. This structural reinforcement is placed at the front of the shoe and is intended to protect the user’s toes from falling objects and compressive loads [5]. The required mechanical performance of each type of protective footwear, according to the ISO standards is given in Table 1.

Traditional safety shoes are made of robust materials, which helps to withstand harsh environments. Although it may increase protection, over-dimensioning can lead to excessive weight and manufacture problems. There are studies that correlate the extra weight and inflexible design of safety shoes with an increase in physical effort (body oxygen consumption is increased) [6,7]. Fatigue together with other factors (uncomfortable and non-aesthetic design, inadequate ventilation) lead workers to reject and/or neglect the usage of this kind of PPE [8,9]. Therefore, the safety footwear market urges for the development of more appealing and comfortable solutions [10].

Historically, steel toe caps started to be manufactured in the early 1920s [11,12,13,14], and they are still used today due to their excellent mechanical properties. Although, it is possible to manufacture thin steel toe caps, its high density makes this component one of the main contributors to the total shoe weight. In most cases, this component can represent up to 35% of the total mass of the shoe, thus contributing to user fatigue and leading to the rejection of this kind of PPE [15].

Nowadays, several types of materials can be used to manufacture toe caps, apart from steel, it is possible to find commercial solutions made of aluminum [16], and non-metallic solutions, such as plastic [17] and plastic-based composites [18]. Non-metallic solutions are highly attractive due to their specific mechanical properties, relative ease of manufacturing, freedom of design and, additional desirable features, such as being non-magnetic and electrical insulators, while providing better thermal insulation than their metallic counterparts [19,20]. Another advantage of plastic toe caps is the ability to recover part of its original shape after imposing a high deformation [21,22]. Despite these advantages, and fulfilling the requirements presented in Table 1, the current non-metallic options are bulky, resulting in clear aesthetic problems [23]. To push new toe cap solutions to the market, research has been advanced on two main fronts: material development and design optimization [24]. 

For material development, some studies have shown an up to 40–56% weight reduction by using fiber-reinforced polymer. Lee et al. developed an optimized stacking sequence layer of glass fiber polyester composite toe cap, with excellent impact and static compression behavior, which is 40% lighter than their steel solution [21]. Yang et al. presented a thermoplastic solution of polypropylene matrix reinforced with sisal fibers, which were modeled and, later, validated with experimental data [19]. The same author also developed a biodegradable solution of flax fibers and PLA for a toe cap, accomplishing a 50% weigh reduction when compared to their steel counterpart, while sustaining the requirements for quasi-static compressive loading according to ISO 12568 [25]. Zukas et al. found a 20% improvement in low velocity impact response, by the addition of nanofillers in carbon fiber reinforced toe caps made of methyl methacrylate resin [26]. More recently, an optimized stacking sequence of E-fiber glass, carbon and aramid fabric layers was suggested by Erden et al. [20]. An overshoe protector that comprises this toe cap alongside a cover made of aramid fabric and TPU resin was also presented, resulting in the creation of a 56% lighter solution than steel counterparts, that were approved in an impact test done with 100 J. Several studies are also reported in the field of metallic toe caps, where different grades of steel and geometries were tested, resulting in an up to 50% thickness and weight saving when compared to their initial designs [15,23,27,28,29]. Although non-metallic solutions present promising results, a number of challenges remain related to material development, design optimization, and speed and ease of manufacturing, while keeping production cost comparable to current metallic solutions.

Design optimization through computational modeling is reported for metallic [15,23,27,28,29,30,31] and non-metallic toe caps [19,21,25], resulting in a reduction of weight by 50% with thickness reduction compared to traditional solutions. Since the introduction of CAD/CAE tools, optimization of product design has been an ongoing pursuit. Even in the niche market of security footwear, commercial software solutions, such as ANSYS LS-Dyna and ABAQUS, have been used to simulate the impact and compressive behavior for toe caps. Both approaches have shown good agreements with experimental results providing a better understanding of stress distribution and resultant deformation, pushing innovation in toe cap design. These software’s have also been reported to be of outmost importance on the analysis and design of safety systems [32,33,34]. However, the use of commercial software for the analysis of dynamic loadings requires a license that can be expensive, and thus, inaccessible to small/medium size companies. Here, it is worth noting that a report from the European Union stated that, in 2012, the footwear industry was heavily populated with small and medium sized companies with 10–15 workers [35]. Acquiring commercial software for these companies is usually prohibitive and, consequently, R&D is primarily performed by trial and error, without the support of CAE. 

In this work, a computational tool based on an open-source computational solver for solid mechanics, built within the OpenFOAM^®^ framework, *solids4Foam* [36], was used to simulate the impact and quasi-static compression behavior of a safety footwear polymer toe cap under the conditions defined in EN 12568 [37]. This manuscript is organized in the following way: in Section 1.2 a brief description of the toolbox used is given. Then, morphological and mechanical characterization performed on a commercial toe cap, kindly provided for this work, is reported (Section 2.1). Subsequently, compression and impact tests performed on the toe cap are described along with the description of the numerical setup within *solids4Foam* to simulate the mechanical tests (Section 2.2). In Section 3, the results from the characterization performed to the material are presented (Section 3.1); a comparison between the results obtained with the component performance evaluated in laboratory testing and the numerical simulations is performed (Section 3.2 and 3.3). After, a mesh convergence study to obtain mesh size independence results will be presented (Section 3.4). Finally, in Section 4, conclusions are drawn about the use of the toolbox for this kind of simulation with special focus on the footwear industry.

### 1.2. Fluid-Solid Interaction within OpenFOAM^®^

Over the last two decades, OpenFOAM^®^ has become a staple for open-source CFD simulations. Its open-source framework based on the object-oriented paradigm allowed several contributions from academia and industry to be implemented leading to substantial improvements in performance and optimization of continuum mechanics solvers. An example of these contributions is the *solids4Foam* toolbox [36], which is a freely available solid mechanics and FSI package distributed via the foam extend fork [38]. The open-source character allows the user to check and adapt the source code to suit its prerequisites. This tool has been used in some works and compared with other industrial commercial software, mostly based on the finite element method [39,40,41,42,43,44]. The results shown are quite impressive, confirming that the finite volume method is a suitable alternative to the conventional finite element one, to solve solid mechanics problems. The toolbox was designed for simulations of solid mechanics and fluid-solid interaction and aims at solving the standard governing equations: conservation of mass, Equation (1), conservation of energy, Equation (2), and conservation of linear momentum, Equation (3) [36].

(1) $$\frac{D}{Dt}\int_{\Omega }^{\;}\rho \;d\Omega =0$$

(2) $$\frac{D}{Dt}\int_{\Omega }^{\;}\rho c_{p}Td\Omega =-\oint_{\Gamma }^{\;}n.qd\Gamma +\int_{\Omega }^{\;}\sigma :\nabla U\;d\Omega$$

(3) $$\frac{D}{Dt}\int_{\Omega }^{\;}\rho U\;d\Omega =\oint_{\Gamma }^{\;}n.\sigma \;d\Gamma +\int_{\Omega }^{\;}\rho b\;d\Omega$$

The symbols used in the above equations are: material density (ρ), cell volume (Ω), cell surface (Γ), normal vector to cell surface (n), specific heat capacity at constant pressure ($c_{p}$), temperature (T), heat flux (q), Cauchy stress tensor (σ), velocity vector (U) and body force per unit mass (b). For solid mechanics analysis, Equation (3) is employed in a Lagrangian form for structural analysis. In the present research it is important to use a solid constitutive law able to describe large deformations and rotations since the compression and the impact events can cause high levels of deformation to the toe cap. At this time, the *solids4Foam* toolbox has some non-linear elastic constitutive laws avaliable, such as: neo-Hookean elastic, St. Venant Kirchhoff elastic, orthotropic St. Venant Kirchhoff elastic; and a non-linear elastic/plastic constitutive law: neo-Hookean elastic with *J*_2_/Mises plasticity [36]. Since the post yield material behavior is an important parameter to consider to adequately simulate the real behavior of the toe cap, the neo-Hookean elastic with *J*_2_/Mises plasticity was chosen. Similar constitutive models were used to study impact tests of different materials [45,46,47,48,49,50]. This law describes the Cauchy (true) stress tensor as follows:

(4) $$\sigma =\frac{1}{J}\left[\mu \;dev\left[B\right]+\frac{K}{2}\left(J^{2}-1\right)I\right]$$

where μ is the shear modulus, $dev\left[B\right]$ is the deviatoric component of the left Cauchy-Green deformation tensor, K the bulk modulus, J the Jacobian of the deformation gradient, and I the second order identity tensor.

## 2. Materials and Methods

### 2.1. Material Characterization

Commercially available thermoplastic toe caps used in safety footwear were kindly provided by a company partner for this work. Several characterization techniques were used, Fourier Transform InfraRed spectroscopy (FTIR), Differential Scanning Calorimetry (DSC) and Scanning Electron Microscopy (SEM) to identify the material type, and quasi-static uni-axial tensile tests were performed to obtain the stress-strain behavior of the material.

#### 2.1.1. InfraRed Spectroscopy

FTIR analysis of a film from the toe cap material with a thickness of ca. 10 μm, prepared using a small hydraulic press heated at 200 °C under a compression force of 10 tons for 30 s, was used to identify the type of polymeric material. The analysis was performed in a Jasco 4100 spectrophotometer (JascoInc., Easton, MD, USA) in transmission mode in the wavenumber range of 400–3500 cm^−1^, with 2 cm^−1^ resolution and an accumulation of 32 spectra.

#### 2.1.2. Thermal Analysis

The thermal behavior of the material was analyzed by DSC, in a Netzsch 200 Maya (Netzsch, Germany), under a nitrogen atmosphere with a heating rate of 10 °C/min from 30 to 200 °C. To eliminate the thermal history of the material, two scans were performed. The results shown in this work refer to the second scan only.

#### 2.1.3. Morphology

The material morphology was assessed by SEM analysis, using an FEI Quanta 400 (FEI, Eindhoven, The Netherlands), samples were previously fractured in liquid nitrogen and coated with a thin gold-palladium film.

#### 2.1.4. Mechanical Characterization

To evaluate the toe cap’s mechanical properties, several toe caps were frozen in liquid nitrogen and fractured with a hammer. The material was subsequently ground to obtain granules of appropriate size for processing. Finally, a dog-bone type 1A specimens according to ISO 527 (gauge length of 50 mm and a nominal cross section of 10 × 4 mm) were prepared by injection molding. Five specimens were used to test the material according to ISO 527 specifications, with a crosshead velocity of 50 mm/min, on an Instron 5969 (Instron, Norwood, MA, USA) with an optical extensometer. Elastic and post yield properties were taken from the tests. The elastic modulus was determined from the slope of the initial linear relationship of the stress vs. strain curve. For the post-yield behavior, the definition of a “true” stress in ASTM D 638 was considered assuming a homogeneous deformation of the specimen cross-section, following Equations (5) and (6).

(5) $$\sigma_{Hom}=\sigma_{Eng}\;\;\left(1+\varepsilon_{Eng}\right)$$

(6) $$\varepsilon_{true}=ln\left(1+\varepsilon_{Eng}\right)\;$$

where *σ_Eng_* is the engineering stress, defined as the ratio between the recorded force (*F*) and the initial specimen cross-sectional area (*A*_0_). *Ε_Eng_* is the engineering strain, defined as the ratio between the recorded grip displacement (*dl*) and the initial distance between the grips (*l*_0_). This curve will be named homogeneous tensile curve and will be taken as a more representative description of the material’s stress-strain behavior that will be used to describe the non-linear material behavior as multi-linear isotropic hardening in the hyper-elasto-plastic mechanical law.

### 2.2. Simulation Setup

#### 2.2.1. Toe Cap Geometry

**Figure 1:**
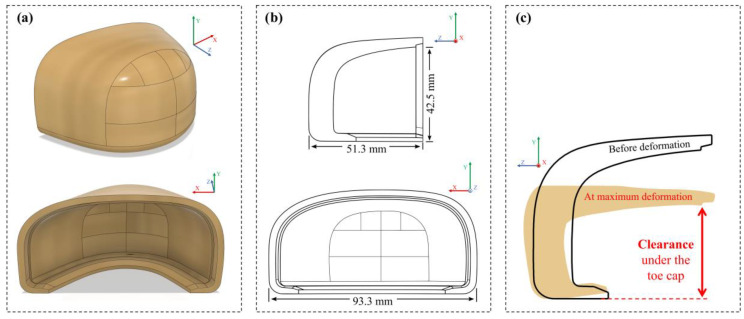
(**a**) 3D front and back view and (**b**) 2D cross-section and back view with general dimensions of the scanned left side size 10 toe cap; (**c**) cross-section view before deformation and at the maximum deformation.

The toe cap geometry present in Figure 1a,b was scanned from a left side size 10 provided by the company partner. In agreement with ISO 20345 and EN 12568 standards, while for non-assembled a minimum clearance under the toe cap of 22 mm is required, after being assembled the value should be greater than 15 mm. At the maximum deformation after a compression or an impact event, the clearance is defined as the remaining height between the base and the inner part of the toe cap at the center (Figure 1c). This parameter is very important to prevent the user’s feet to get damage after a compression or an impact event. Some general dimensions regarding the toe cap are provided in Table 2.

For analysis purposes, the value 42.5 mm of the inner height of the toe cap ($h_{i}$) will be used to estimate the clearance value after each simulation. In laboratory, the clearance is measured using a cylinder clay placed under the toe cap. The clay deformation ($h_{clay}$) is equal to the inner height plus the toe cap inner height variation ($\Delta h_{i}$), Equation (7).

(7) $$h_{clay}=h_{i}+\Delta h_{i}$$

#### 2.2.2. Mesh Sensitivity Analysis

To get grid independent results, an interactive mesh convergence study was performed using progressively finer grids, increasing the number of cells 3-fold with each mesh. This study comprised five different meshes with 25 k, 76 k, 228 k, 671 k and 2 M cells. The computational grids were generated with the utility *cfMesh*, a library for automatic mesh generation that is compatible with OpenFOAM^®^, using the Cartesian mesh subroutine [51]. To assess the refinement effect, the von Mises Stress ($\sigma_{Eq}$) and the cell displacement along the *y*-axis ($D_{yy}$) were averaged with a weight corresponding to the cell volume for the overall domain, Equations (8) and (9). Additionally, the variables of interest for the analysis were also tested, such as striker velocity ($U_{y\;striker}$), plate force ($F_{y\;plate}$) and plate displacement ($D_{y\;plate}$), and the toe cap clearance variation ($\Delta h_{i}$), to confirm that grid independent results were obtained. Table 3 displays each mesh cell size and total number of cells.

(8) $$\bar{\sigma_{Eq}}=\frac{\sum \left(V_{cell}\sigma_{Eq}\right)}{\sum V_{cell}}$$

(9) $$\bar{D_{yy}}=\frac{\sum W\left(V_{cell}D_{yy}\right)}{\sum V_{cell}}$$

It is well known that non-orthogonality and skewness are important parameters for simulation stability and reliability in FVM and are often used as a mesh quality indicator [52,53]. A value of zero for the first parameter is an indicator of a fully orthogonal mesh grid, where all grid lines are parallel to each other. Most geometries do not allow the generation of a fully orthogonal mesh, and, therefore, non-orthogonal correction must be implemented to assure the simulation stability and accuracy. Skewness also affects the solutions accuracy, and must be as low as possible to decrease diffusion error during calculation [54]. These parameters should be as low as possible due to the discretization method used to solve the diffusive terms, which uses the normal face vector of each face cell to calculate how fluxes travel through cells. For instance, hexahedron cell types and a more refine mesh tend to have lower average non-orthogonality and skewness values compared with tetrahedral cells, improving the solution accuracy but with a negative impact on computational performance. 

**Figure 2:**
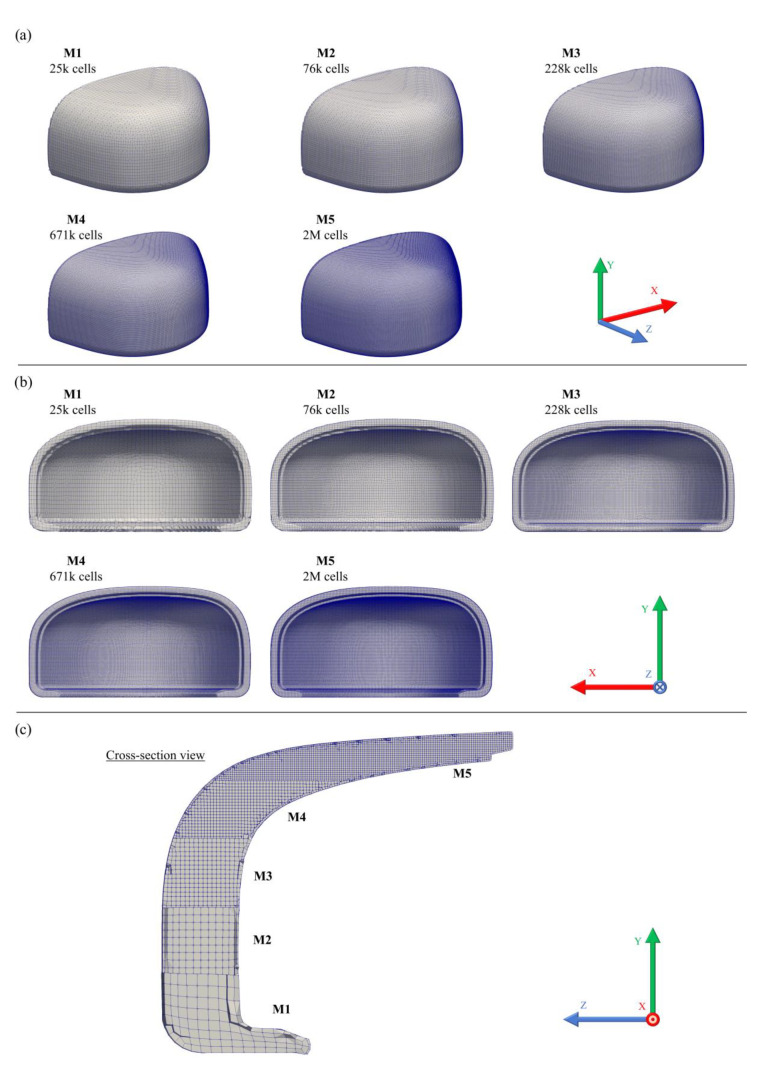
Toe cap meshes (M1 to M5) used on the mesh sensitivity analysis: (**a**) isometric view, (**b**) back view, and (**c**) cross-section view.

All toe cap meshes used for mesh sensitivity analysis are illustrated in Figure 2, with the mesh quality parameters presented in Table 3. In a first analysis, the average cell size decreases and the total number of cells in the mesh increases. Although maximum non-orthogonality and skewness did not decrease with mesh refinement, both average of non-orthogonality and skewness decreases consistently, and, therefore, an improvement in the quality of the mesh is obtained with small cell sizes. For complex geometries, such as toe caps, it is difficult to use just Cartesian cells to generate the mesh near curved surfaces. Therefore, those regions are expected to have cells with lower mesh quality. To prevent simulation problems, this effect can be mitigated by refining the mesh locally. The first approach was used since better mesh results were obtained instead of using local refinements. Figure 2 displays a slice of the vertical section (cross-section view) to show this effect, where a better definition of the geometry is obtained for M4 and M5, especially near the edges/corners, while the interior of the toe cap mesh is dominated by quasi-orthogonal cell. Modeling the toe cap with small cells, the percentage of hexahedron cell type approaches to 100%, in comparison with the remain types of cells (Table 3). 

#### 2.2.3. Quasi-Static Compression Test

**Figure 3:**
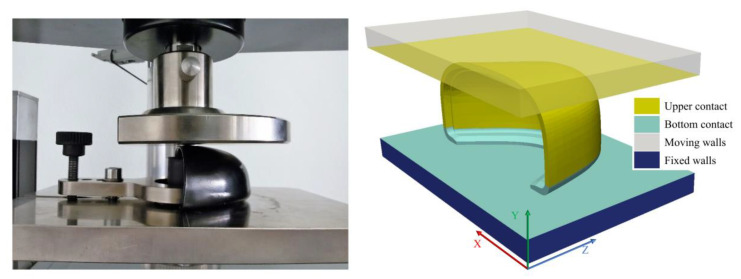
Compression test setup for the safety footwear toe cap-experimental configuration (**left**), computational model with the indication of the boundary patches (**right**).

The most demanding category of footwear is the safety class, according to ISO 20345, this type of footwear must withstand a compressive load of 15 kN while preserving a minimum standardized clearance value, which is dependent on the toe cap size that was measured with the help of a standardized clay cylinder (L = 25 mm, ø = 25 mm) [2]. The test methodology for footwear is defined in ISO 20344 [55], and for the non-assembled toe cap in EN 12568 [37]. The laboratory and the simulated compression test are depicted in Figure 3. To perform this simulation the digitalized commercial polymeric toe cap geometry was modelled and placed between two steel plates, mimicking the experimental setup. The boundary conditions for the components in the simulation were the following:

- the bottom plate was set to have a zero displacement in all Cartesian directions (Fixed Walls patch in Figure 3 Right);
- the top plate was set to have a velocity of 5 mm/min along the negative *y*-axis (Mixed walls patch in Figure 3 Right);
- the toe cap was set as the contact region between the top and bottom plates, using a penalty method for the normal and tangential contact behavior; a Coulomb friction law with friction coefficient of 0.3 [56,57] was used (Bottom and Upper contact patches in Figure 3 Right).

Although the test velocity is small, the simulation was performed considering inertial effects. The discretization schemes for each term in the governing equation and the solver control parameters are presented in Table 4. A time step of 0.5 s was chosen and the simulation was allowed to run until the force showed on the top plate (upper contact patch in Figure 3 right) reached a value of 15 kN.

One of the advantages of using OpenFOAM^®^ is the possibility of parallelizing the calculation. Since there are no limits associated to licenses, parallelization is only constrained by the accessible hardware. For this simulation, the computational domain was divided into eight physical processors (2.6 GHz and 2 Gb of RAM) using the Metis decomposition method and ran on a computational cluster using one node.

#### 2.2.4. Impact Test

**Figure 4:**
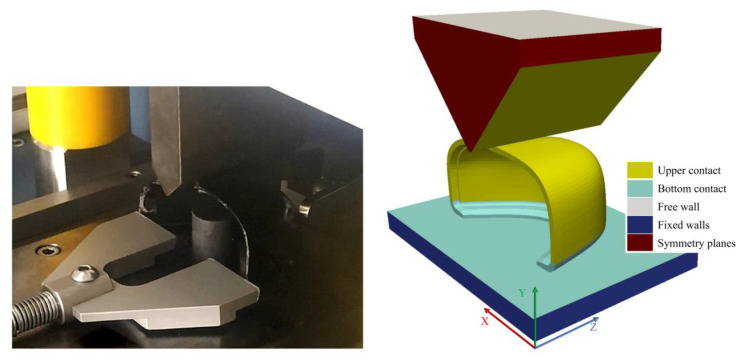
Impact test setup for the safety footwear toe cap: experimental configuration (**left**), computational model with the indication of the boundary patches (**right**).

Toe caps used in safety footwear must withstand an impact loading of 200 J, according to ISO standards, the impact behavior is accessed by measuring the height of a standardized cylinder clay before and after the test. These values must be within a specified clearance range during product validation, as shown in Table 2. For the geometry used in this work, the toe cap should withstand the impact event with a clearance higher than 22.0 mm. ISO 20344 defines that the impact test must be performed with a 20 kg steel striker positioned at a predetermined height in order to achieve the required impact energy during the free fall [55]. For simulation purposes these conditions were met by modeling the impact striker geometry as defined in ISO 20344, placing it close to the top surface of the toe cap, and adjusting its velocity to assure the required impact energy [55]. After designing the striker and considering the density of steel at ambient temperature (7850 kg/m^3^), the final weight was 1.547 kg and the resulting velocity set to 16.081 m/s to assure the impact energy requirement. The case setup and the simulated scenario are depicted in Figure 4.

In this simulation the inertial effects are of uttermost importance, the numerical schemes and solver controls were the same used on the compression simulation with the addition of a relaxation factor of 0.95 applied to the computational fields, a time step of 8 × 10^−6^ s was chosen, and the simulation ran until the striker velocity along the *y*-axis reached a value of 0 m/s. The boundary conditions for the components in the simulation were as follows:

- The base plate was defined to have a fixed zero displacement in all Cartesian directions (Fixed walls patch in Figure 4 Right);
- To constrain the striker to the y-direction, the faces with normal vector pointing into x- and z-direction were set as symmetry planes (Symmetry planes patches in Figure 4 Right);
- The contact regions were simulated with the same method and values used for the compression structural analysis (Upper and Bottom contact patches in Figure 4 Right).
- The initial velocity was imposed on the striker by using the OpenFOAM ^®^ utility, *setFields*.

## 3. Results and Discussion

### 3.1. Toe Cap Material Characterization

**Figure 5:**
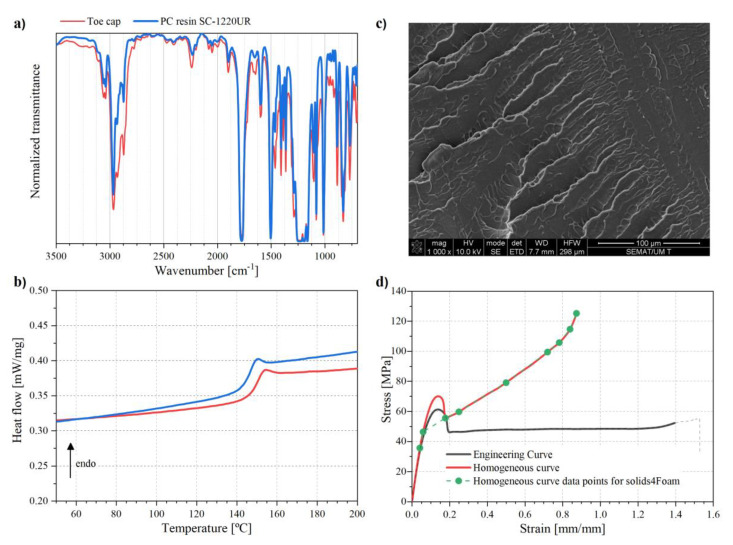
Toe cap material characterization: (**a**) FTIR spectrum, (**b**) DSC thermogram, (**c**) SEM image and (**d**) tensile test curve.

Figure 5a presents a FTIR transmission spectra of the sample from the toe cap and polycarbonate (PC) grade INFINO SC-1220UR. The sample from the toe cap shows several intense peaks at 1776 cm^−1^, 2968 cm^−1^, 2873 cm^−1^, and the doublet at 3060 and 3042 cm^−1^, where the first corresponds to the carbonyl stretching, and the others assigned to the aromatic bisphenol structure, these peaks are characteristic of a PC thermoplastic [58]. To confirm, a sample of PC available in our laboratory was also prepared under the same conditions and analyzed. All peaks are at the same position, which confirms that the toe cap material was mainly PC.

The DSC test was performed to check the glass transition (T_g_) of both materials. Figure 5b demonstrates that both have a single T_g_ around 150 °C, which is coincident with known T_g_ values reported in literature for amorphous PC [59].

The surface morphology presented in Figure 5c does not show any particles in the polymer matrix, in fact, only one phase can be detected, which is characteristic of a neat polymer. Through the previous analysis it is possible to infer that the toe cap material is composed of neat PC without any reinforcements or other polymer added.

Figure 5d illustrates the engineering (black line) and the calculated homogeneous tensile curve (red line). The material displays strain softening due to yielding and neck formation at a strain between 0.05 and 0.175. Following this, a strain hardening effect can be observed due to the orientation of the macromolecules along the load direction until the specimen fracture. For the simulation studies, the material was defined as neo-Hookean elastic, with the elastic limit defined as the end point of the linear relationship between σ-ε on the homogeneous curve. The green dot symbols, Figure 5d, are the points added to the constitutive model as a multi-linear isotropic hardening used in the computational studies. Conventionally, the yield stress is the transition point between elastic and plastic domain, but it is known that polymeric materials present some plastic deformation for lower stresses. Therefore, for simulation purposes, yield stress point was chosen to be the value where stress-strain curve loses its initial linearity, around 35 MPa.

Additional data for the constitutive model employed on the simulations are presented in Table 5, together with the linear elastic model data considered for the plates and striker parts.

### 3.2. Mesh Sensitivity Analysis

**Figure 6:**
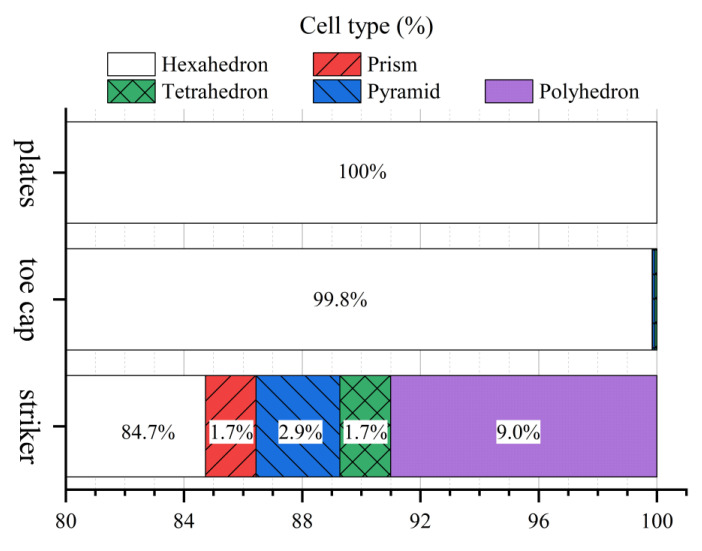
Percentage of cell type distribution.

**Figure 7:**
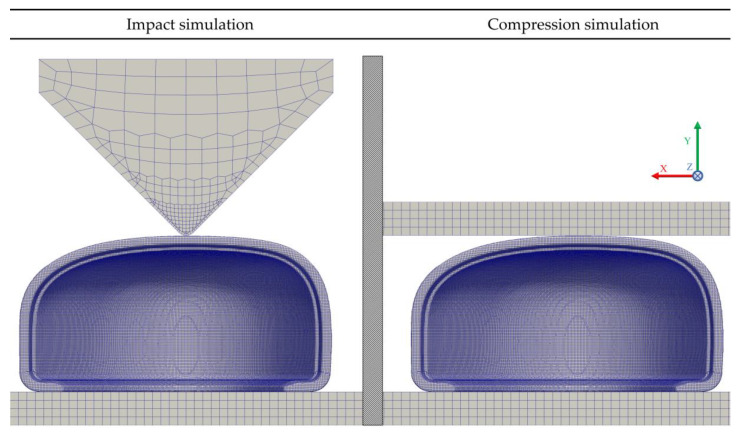
Back view, M4 and plates mesh for the impact and the compression simulation cases.

The mesh quality information regarding the setup cases for impact and compression simulations using the M4 toe cap mesh are presented in Table 6 and Figure 6. As discussed in Section 2.2.4, the toe cap mesh is mainly comprised of hexahedral cells, where meshes M4 and M5 have the lowest average non-orthogonality. Additionally, due to its simple (cubic) geometry both plates (bottom and top) are comprised of just hexahedral cells. The local refinement of the striker mesh at the impact zone leads to a high level of non-orthogonality, with more than 15% of non-hexahedral cells. Despite the high non-orthogonality value for the metallic parts, simulation errors are not expected, since both the striker and plates have a significant higher elastic modulus than the toe cap, which extensively reduces these parts deformation. Figure 7 displays the setup cases with the generated meshes for the M4 mesh.

**Figure 8:**
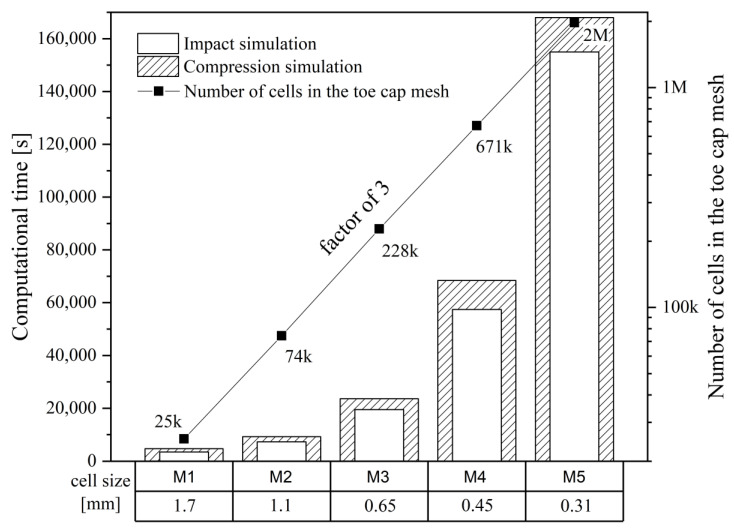
Details of the mesh study for both mechanical analyses.

An overview of the mesh sensitivity study, Figure 8, shows an expected exponential increase in computational time with increasing number of cells. While for the coarsest mesh (M1), it takes around one hour to complete each simulation, for the finer mesh (M5) almost two days are required. It is also visible that, until the simulation reaches the point of interest, the compression case is a little bit more time consuming for the same hardware resources.

**Figure 9:**
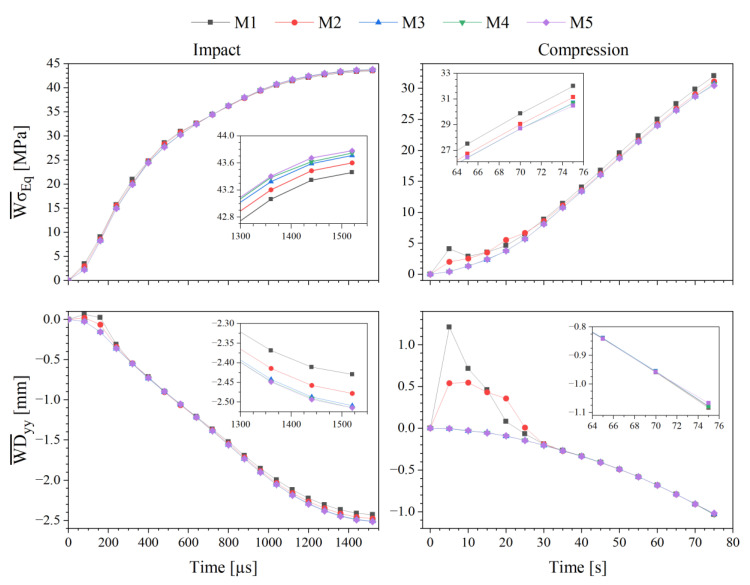
Analysis of the average von Mises stress and y-displacement evolutions as a function of mesh size for impact and compression simulations.

**Figure 10:**
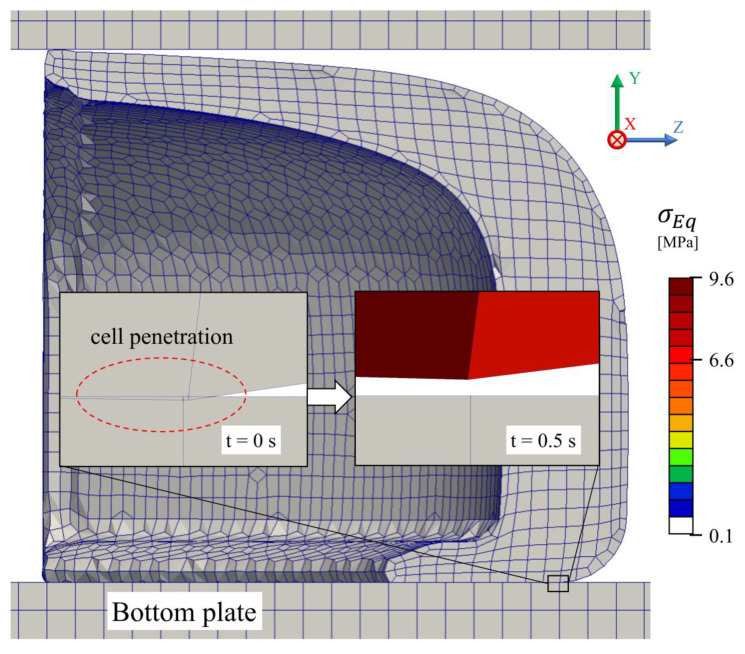
Induced tension due to cell penetration with M1 toe cap mesh in compression simulation for 0 s and 0.5 s.

To check for grid convergence, the overall domain variable distributions were averaged by the ratio between a property 𝜙 and the cell volume. The results for the compression and impact tests are presented in Figure 9. In both cases, the weighted (Equations (8) and (9)) von Mises stress and displacement along the *y*-axis are plotted and, as expected, when the mesh is refined the results converge. For coarse meshes an enormous variation in values is visible for the initial time steps. In geometries with round edges, the mesh generation can create vertices that might be outside the geometry boundaries. In this case, for meshes M1 and M2, there were some cells from the bottom of the toe cap that intersect the bottom plate cells (Figure 10), resulting in induced stresses at the beginning of the simulation. The resultant induced stresses were in the elastic domain of the toe cap material. Therefore, no plastic deformation is induced due to cell crossover. From these results, it is also clear that the weighted values do not have a significant variation between meshes M4 and M5.

**Figure 11:**
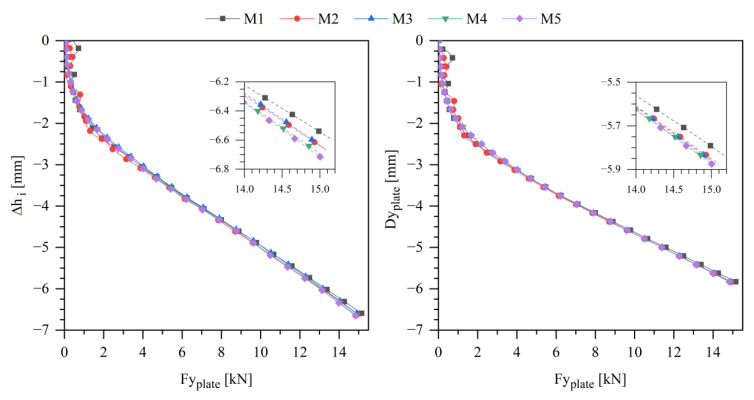
Inner toe cap y-variation and top plate y-displacement plot over plate y-force, for compression simulation.

The compressive simulation results of $\Delta h_{i}$ and top plate displacement ($Dy_{plate}$) as a function of the top plate *y*-resultant force ($Fy_{plate}$) are depicted in Figure 11. The insert indicates that $\Delta h_{i}$ is a little bit more sensitive to mesh refinement than $Dy_{plate}$. Between the coarsest and finer meshes, a 0.2 mm difference of $\Delta h_{i}$ value is reported.

**Figure 12:**
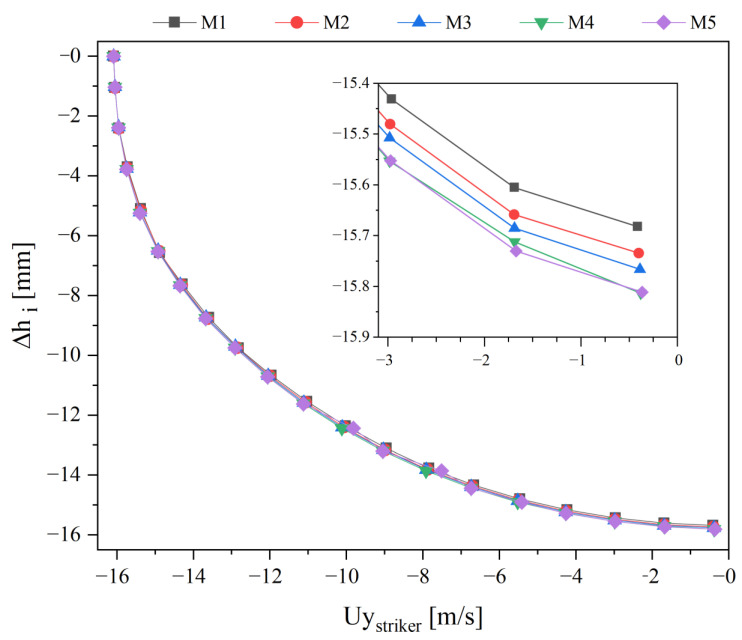
Inner toe cap y variation vs. striker velocity, for impact simulation.

Figure 12 exhibits the effect of mesh refinement over the inner toe cap wall displacement as a function of the striker velocity in the *y*-direction. A tendency for convergence in the maximum $\Delta h_{i}$ value when the impact stops (Uy = 0 m/s) is noticed. Moreover, the M5 mesh predictions are similar to the ones for M4 mesh. A difference of 0.13 mm is seen between the M1 and M5 for the final $\Delta h_{i}$, which indicates that the cell crossover at the initial time steps just affect the compression simulation, on the impact counterpart there are no visible effect.

**Figure 13:**
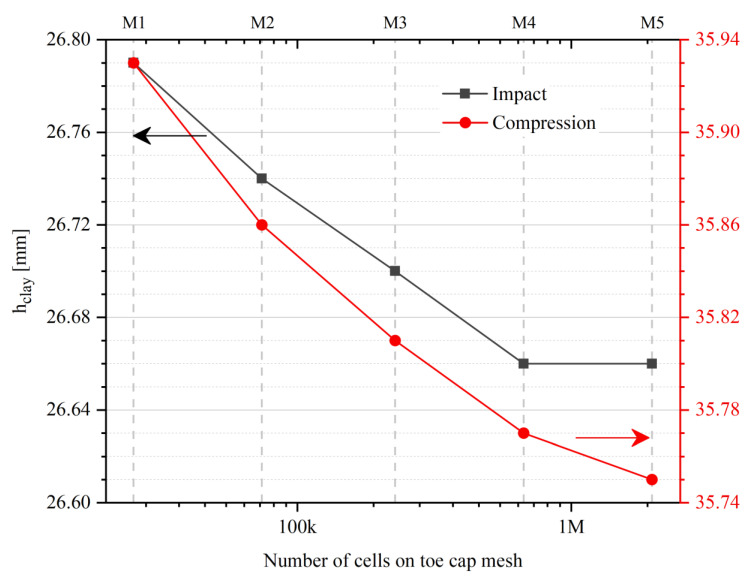
Clay height for impact (left axis) and compression (right axis) simulations as a function of number of cells on toe cap mesh.

To quantify the practical impact of mesh refinement in the estimated clay height values, Figure 13 plots the $h_{clay}$ value for impact (left axis) and compression (right axis) simulations as a function of the total toe cap cell number. For the impact simulation there is no value variation between M4 and M5, while for the compression case a lower value of variation is seen for the two most refined meshes.

Based on these results, for the remaining analyses it was decided to use mesh M4 since it is the one that presents the best balance between precision and computational cost.

### 3.3. Quasi-Static Compression Test

**Figure 14:**
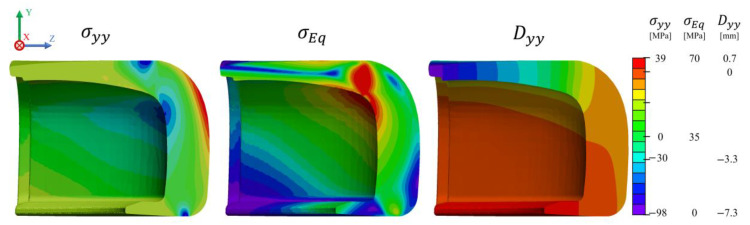
Normal stress ($\sigma_{yy}$), von Mises Stress ($\sigma_{Eq}$ ) and cell y-displacement ($D_{yy}$ ) distribution inside the toe cap for compressive simulation, when $Fy_{plate}=15\;\mathrm{kN}$.

During the compression loading simulation, the force on the loading plate was monitored until reaching a value of 15 kN. Afterwards, Paraview v5.6.2 was used for post-processing of the simulation data [60]. The results from the normal stress field along the *y*-axis ($\sigma_{yy}$), von Mises Stress ($\sigma_{Eq}$) and *y*-displacement ($D_{yy}$) are depicted in Figure 14. Analysis of the contour plots indicates the existence of a complex state of stress within this part. Regarding $\sigma_{yy}$, a maximum compressive stress around 100 MPa is located at the top and bottom of the toe cap, at the contact regions between the toe cap and the top and bottom plates, whilst a tensile stress close to 40 MPa is identified at the top front of the toe cap. A negative *y*-displacement is higher at the top back of the toe cap, which contributes to the final value of $h_{clay}$, while the rest of the toe cap is pushed up as a result of the resultant force on the bottom plate. The von Mises stress on the cross-section is displayed in Figure 14, revealing that the contact points are critical areas where the material will have a higher plastic deformation. Furthermore, the inner material seems to be more protected from the resultant stresses than the outer material. Consequently, a higher degree of permanent deformation at the top region of the toe cap is expected, where von Mises stress is higher than 70 MPa. Furthermore, $\sigma_{yy}$ shows that the toe cap presents some bending and stretching at the toe cap surface (positive values), and compression at the inner side (negative values).

**Figure 15:**
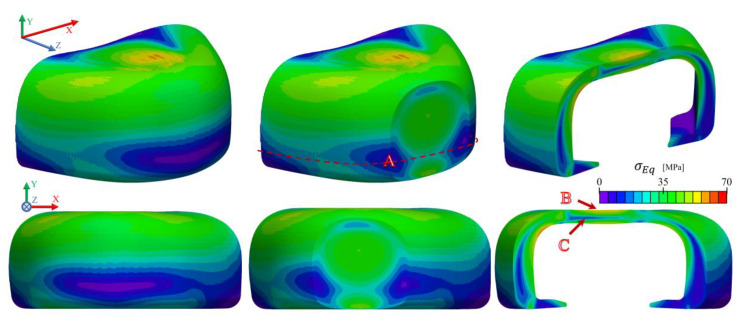
von Mises stress distribution in the compressive simulation computed with the M4 toe cap mesh.

To better understand how the von Mises stresses evolve inside the toe cap, Figure 15 demonstrate the distribution on some slices at different critical regions. It is possible to notice that the thickest region (A) presents uniform stress distribution along the thickness, whose magnitude increases with the thickness reduction, which is typical of axial stress loading, in this case compressive. Progressing to the top, the stress distribution along the thickness changes to bending, with a stress magnitude higher near the surface (B) and lower at the core region (C). These results indicate that in terms of compression, the thicker region could be thinner, since the stress magnitude at the thickest region is always lower than the one induced by bending.

**Figure 16:**
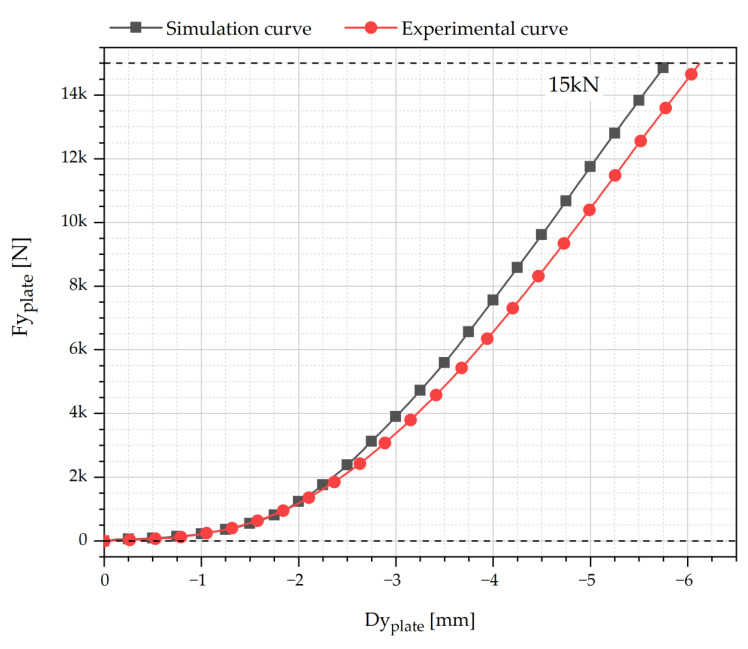
Comparison of the compression behavior of the tested and simulated toe cap.

A comparison between the Force-displacement (*F-d*) curve resulting from the compression test and the one obtained numerically is made in Figure 16. A similar trend for the same curve was obtained in Dirksen et al. [31] and Costa et al. [15]. In the *F-d* curve of the tested toe cap, the maximum loading displacement recorded at 15 kN was 6.12 mm and for the simulation curve was 5.79 mm. As it can be seen, the computational and experimental curves have a good correlation, exhibiting a maximum error of 5.4%.

The obtained results indicate that *solids4Foam* can simulate accurately the compression behavior of a plastic toe cap.

### 3.4. Impact Test

**Figure 17:**
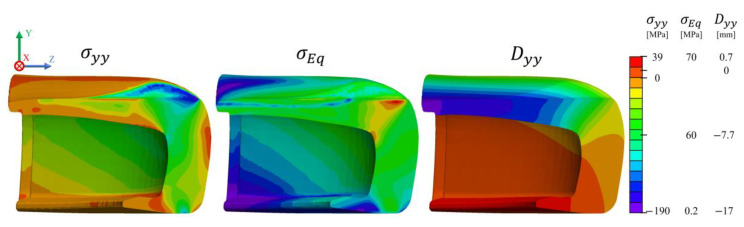
Normal stress ($\sigma_{yy}$), von Mises Stress ($\sigma_{Eq}$ ) and cell y-displacement ($D_{yy}$ ) distribution in the toe cap for the impact simulation, when $Uy_{striker}=0\;m/s$.

**Figure 18:**
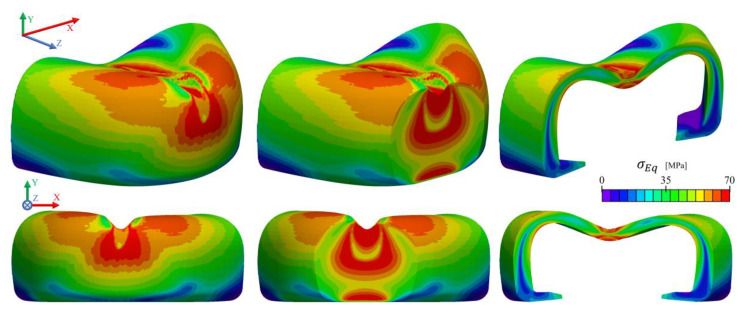
von Mises stress distribution on impact simulation of M4 toe cap mesh.

Figure 17 presents the stress and displacement fields along the *y*-axis obtained in the impact test, at the moment of maximum displacement of the striker (where it reaches a zero velocity). The highest compressive stresses (negative $\sigma_{yy}$) are located at the bottom contact regions of the toe cap, between the striker and the bottom plate, with a maximum value of approximately 200 MPa. The remainder of the part is mainly under compression (negative $\sigma_{yy}$), with certain regions presenting tensile stress (red zones at Figure 17 for $\sigma_{yy}$) near the surface. It is possible to deduce a complex state of stress along the cross section of the toe cap, where a strong compressive behavior is observed at the top and propagates in a wave-like form from the point of impact to the base support (Figure 17 $\sigma_{Eq}$). This behavior is clearly visible in Figure 18, where slices at some critical regions are shown with von Mises stress values to better identify yielding spots. From these results, one can realize that the impact zone (region where the striker hits the toe cap) is clearly the most severely affected region and will be the zone with the highest plastic deformation. When compared to the compression test simulation, the impact event more aggressively causes higher von Mises stresses, reaching a maximum value of 120 MPa at the impact region. From the images, it is possible to assume that during the impact test the principal way to accommodate the impact energy is through compression and bending. A thin shell of higher stress values is also visible for the thinner parts of the toe cap. 

**Figure 19:**
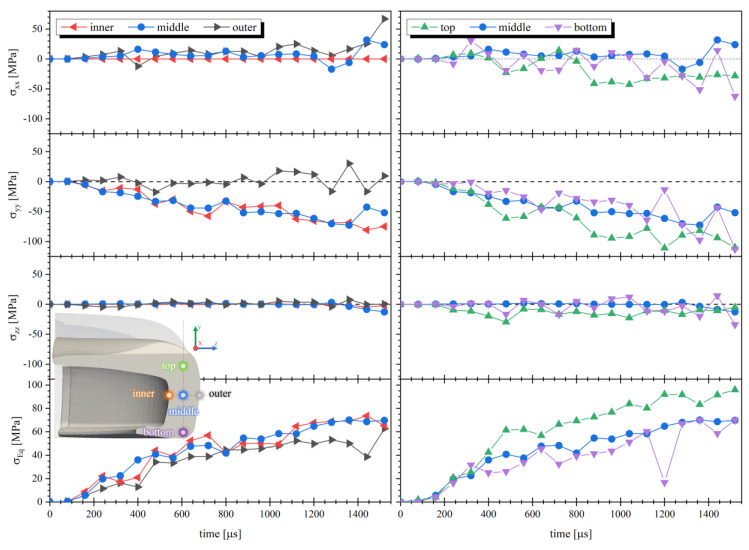
Stress at xx ($\sigma_{xx}$), yy ($\sigma_{yy}$ ) and zz ($\sigma_{zz}$ ) direction, and von Mises Stress ($\sigma_{Eq}$ ) evolution at the inner, middle and outer (left), top and bottom (right) of the toe cap horizontal and vertical y-z plane.

Figure 19 displays the tension evolution in five points (inner, middle, outer, top and bottom) along the toe cap thickness and the height at the y-z plane during simulation time, and the wave-like propagation of $\sigma_{Eq}$ visually observed in Figure 18. Bending is also detectable in the inner and outer points having negative and positive $\sigma_{yy}$ values, respectively, while in the vertical direction all points have a negative value (compression). $\sigma_{yy}$ and $\sigma_{xx}$ are the stress components that contribute the most for the stress state of the toe cap, while the $\sigma_{zz}$ contribution has a negligible effect at the analyzed zone.

Since no boundary was placed to restrict the movement of the base of the toe cap, due to the rebound that occurs in the test (which is also observed experimentally), it is possible to observe that the base presents a positive *y*-direction displacement of 1.7 mm (Figure 17 $D_{yy}$). The displacement of the top region is quite pronounced along the negative *y*-direction, as would be expected due to the nature of the test, reaching a maximum value of 17 mm.

**Figure 20:**
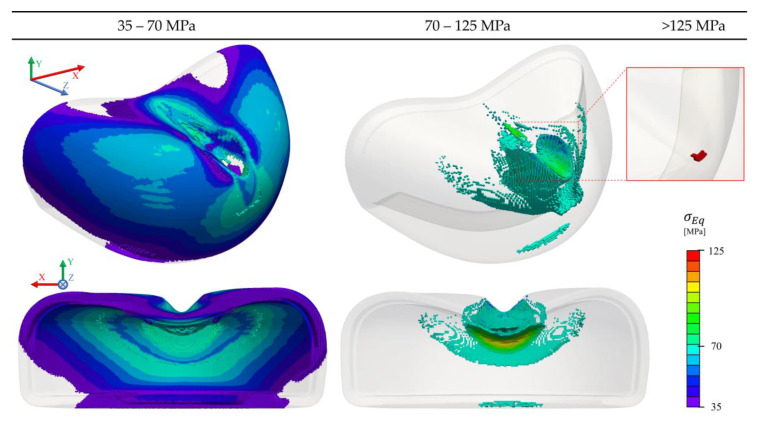
Von Misses stress distribution with applied threshold of (**left**) 35–70 MPa, (**middle**) 70–125 MPa, and (**right**) cells with maximum stress, for the impact simulation.

Based on the achieved results a large number of cells permanently deform due to the high stresses developed. A visibility filter based on $\sigma_{Eq}$ was applied to the results provided in Figure 20, to demonstrate: (a) the cells where plasticity criteria started to be applied (≥35 MPa) and (b) the critical cells which overpassed the peak yield stress (≥70 MPa). It is clear that the yielded region is located at the top of the toe cap, with the remaining areas just deforming in its quasi-elastic region (35–70 MPa). This is an indicator of the most critical zones that needs to be reinforced, as well as the zones that are required to be properly dimensioned. Similar conclusions were obtained in Costa et al. [23], but for steel toe caps. At the maximum strike displacement, where the velocity is null, a total of eleven cells reached the stress at break of the characterized material (125 MPa), which could be associated with rupture. Since no rupture criterion was employed in this simulation, after reaching the maximum stress value defined in the constitutive model, the solver clamps to the last stress value, imposing it for larger deformations.

Finally, in the impact simulation a value of 26.66 mm for the toe cap clearance was obtained. Comparing with a value of 28.6 mm obtained experimentally, the simulation results have a difference of 6.8%. This approach validates the simulation tool, showing good agreement with experimental results, enabling its usage in the design of new toe cap solutions for safety footwear.

**Figure 21:**
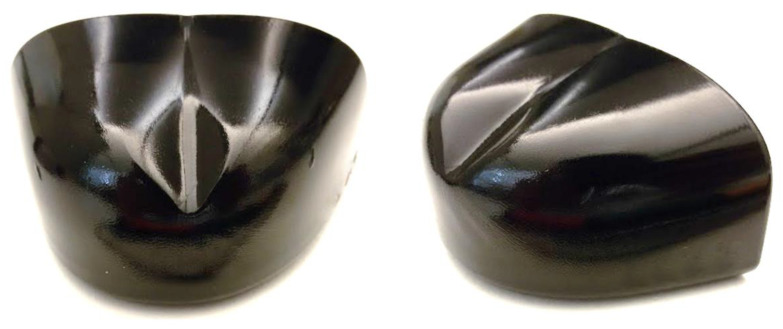
Geometry of a toe cap subjected to the impact test.

The good quality of the numerical results can be further assessed by comparing the final geometry of the toecaps after the impact test, illustrated in Figure 21, which is similar to the final geometries predicted by the numerical code, as shown in Figure 18 and Figure 20. The final geometry is also similar to the ones reported on the impact tests of toe caps performed in Kropidłowska et al. [61].

## 4. Conclusions

This work aimed at assessing the capability of using the *solids4Foam* toolbox, a free and open-source code developed in the framework of the OpenFOAM^®^ computational library, to support the design of toe caps. The characterization of a commercial toe cap allowed the identification of the material typology as a neat polycarbonate without reinforcements, while the mechanical characterization described the stress-strain behavior to be used as input in the numerical simulations of compression and impact tests.

A detailed mesh refinement study was carried out to obtain grid independent results, showing that the final deformation of the toe cap converges to a value by decreasing the cell size. The values acquired for compression and impact simulation revealed a good agreement with the ones obtained in experimental testing, with an error of 5.5% and 6.8%, respectively. These simulations indicated that the impact test is the most demanding (higher stress values) for this type of component. Therefore, it should be used for dimensioning the toe cap design. Critical zones were identified at the contact point between the upper zone of the toe cap and the plate/striker. The stresses developed during the impact tests are distinct from those in the compression tests, exhibiting a wave-like propagation form from the top to the bottom. 

Given the achieved results, it was clearly demonstrated that *solids4Foam* toolbox can offer a significant support in future R&D in the footwear industry. It is important to notice that due to the inherent simplifications associated to the numerical models, the computational methodology is not expected to fully replace the currently experimental trial-and-error design approaches. However, the number of trials performed experimentally are expected to be substantially reduced when the toe cap design is supported with the methodology proposed in this work. Moreover, the possibility of resorting to numerical tools allows performing much more trials, which can clearly contribute to further improve the final toe cap performance.

## Figures and Tables

**Table 1:** Mechanical requirements for each type of protective footwear [2,3,4].

Category of Footwear			
Toe Cap Requirements	Safety ISO 20345	Protective ISO 20346	Occupational ISO 20347
Impact energy (J)	200	100	-
Compression load (kN)	15	10	-

**Table 2:** Minimum clearance value after impact and compression as a function of the size of toe cap and inside the safety footwear [2,37].

**Toe cap size**	≤5	6	7	8	9	≥10
**Toe cap clearance (mm)**	19.5	20.0	20.5	21.0	21.5	22.0
**Shoe size**	≤36	37–38	39–40	41–42	43–44	≥45
**Toe cap clearance inside the shoe (mm)**	12.5	13.0	13.5	14.0	14.5	15.0

**Table 3:** Toe cap meshes properties.

Mesh Properties/Cell Type (Number/%)	M1	M2	M3	M4	M5
cell average size (mm)	1.7	1.1	0.65	0.449	0.31
Total number of cells	25,216	74,272	227,541	670,640	1,973,199
Max. non-orthogonality	64.16	67.75	61.40	57.11	58.69
Average non-orthogonality	19.28	15.6	3.98	3.15	2.61
Max. skewness	1.12	0.99	1.22	1.86	0.99
Average skewness	0.29	0.22	0.09	0.07	0.05
Hexahedron	24,598 (97.55%)	73,243 (98.61%)	226,626 (99.60%)	669,560 (99.84%)	1,971,480 (99.91%)
Prism	56 (0.22%)	74 (0.10%)	98 (0.04%)	136 (0.02%)	206 (0.01%)
Pyramid	258 (1.02%)	409 (0.55%)	383 (0.17%)	472 (0.07%)	715 (0.04%)
Tetrahedron	304 (1.21%)	546 (0.74%)	434 (0.19%)	472 (0.07%)	798 (0.04%)

**Table 4:** Discretization schemes and solver control parameters.

Discretization Schemes		Solver Control Parameters	
d2dt2	Euler	D, DD and sigmaHyd	
Time	Euler		
Gradient	Least squares	Solver	PCG
Divergence	Gauss Linear	Preconditioner	FDIC
Laplacian	Gauss Linear corrected	Tolerance	1 × 10^−7^
laplacian(DD,D)		Relative tolerance	0.1
laplacian(DDD,DD)			
Surface normal gradient	New skew corrected 1		
snGrad(D)			
nGrad(DD)			
Interpolation	Linear		

**Table 5:** Mechanical properties of the materials used for simulation.

	Toe Cap	Plate/Striker	
Elastic modulus	2.5	200	GPa
Poisson ratio	0.3	0.3	-
Density	1200	7850	kg/m^3^
Initial yield stress	35.7	-	MPa

**Table 6:** Relevant quality parameters of the used meshes.

Mesh Size and Additional Information			
Component	Striker	M4	Plates
Nr. of elements	8168	670,640	8208
Max. aspect ratio	7.49	7.13	1.56
Non orthogonality:			
Maximum	62.81	57.11	2.16
Average	18.42	3.15	0.56
Max. skewness	0.63	1.86	0.04

## Data Availability

The data presented in this study is available in the article.
